# Baohuoside-I suppresses cell proliferation and migration by up-regulating miR-144 in melanoma

**DOI:** 10.1080/13880209.2017.1418391

**Published:** 2017-12-20

**Authors:** Ya-Guang Peng, Li Zhang

**Affiliations:** Shandong Provincial Hospital affiliated to Shandong University, Jinan City, Shandong Province, China

**Keywords:** Antitumour effects, SMAD1, invasion

## Abstract

**Context:** Baohuoside-I was reported to induce apoptosis in non-small-cell lung cancer and inhibit the growth of multiple myeloma cells. The antitumour potential of baohuoside-I has not been demonstrated in melanoma yet.

**Objective**: To investigate the potential antitumour activity of baohuoside-I against melanoma and elucidate its underlying molecular mechanism.

**Materials and methods:** Cell viability was evaluated by MTT assay. The malignant invasion capacity was measured with trans-well assay. The relative expression change of microRNAs was profiled with microarray. TargetScan was utilized for prediction of target gene of miR-144. Regulatory effect of miR-144 on SMAD1 was determined by dual luciferase reporter assay. Endogenous SMAD1 protein in response to ectopic expression of miR-144 was determined by immunoblotting. Xenograft mice were employed to evaluate antitumour potential of baohuoside-I (25 mg/kg by tail intravenous injection every two days) *in vivo*.

**Results:** Baohuoside-I significantly inhibited proliferation (45 ± 4% reduction in M14 and 35 ± 3% reduction in MV3 at 24 h) and migration (70 ± 4% reduction in M14 and 72 ± 3% reduction in MV3) in melanoma cells. Mechanistically, baohuoside-I up-regulated miR-144 expression levels (3 ± 0.2-fold). Silence of miR-144 reversed the inhibition of baohuoside-I in melanoma. We have identified that SMAD1 was the novel target of miR-144. Moreover, baohuoside-I suppressed melanoma *in vivo* (52 ± 8% reduction in xenograft tumour size at day 20).

**Conclusions:** Our data suggested significant antitumour potential of baohuoside-I against melanoma both *in vitro* and *in vivo*, which warrants further laboratory investigation and clinical trial.

## Introduction

Melanoma is a human malignance arises from melanocyte, which physiologically produces melanin (Miller and Mihm 2006). Melanoma is the most serious type of skin cancer, which also occur in the mouth, intestines, or eyes but relatively rare. Worldwide, 232,000 new cases were diagnosed in 2012, and 59,800 deaths were claimed by this disease in 2015 (GBD 2015; Mortality and Causes of Death Collaborators 2016). The well-established risk factors associated with melanoma include Caucasian race, fair skin, intensive sunlight exposure and genetic mutations (Rhodes et al. 1987). Excessive ultraviolet light (UV) exposure is the primary cause of melanoma in those with low abundance of skin pigment, therefore is preventable by sunscreen and avoiding UV light (Wang et al. 2001). Clinical diagnosis of melanoma is feasible through visual inspection; biopsy can be performed for confirmatory purpose (Kozovska et al. 2016). Moles characterized with typical symptoms and signs as described in conventional term ABCDE (Asymmetrical skin lesion, Border irregularity, Colour change or multiple colours, Diameter more than 6 mm, Evolves over time) warn of following regular examinations (Friedman et al. 1985). Early-diagnosed melanoma is curable by complete surgical excision with adequate surgical margins. For those advance malignant melanoma, comprehensive and multidisciplinary approach should be applicable, such as chemotherapy, radiotherapy and immunotherapy (Singh et al. 2017). However, the clinical benefit of these regimens is very limited for metastasized melanoma, the 5-year survival rate of which is between 7 and 19%. Therefore, it is still in urgent need for finding new target and novel therapeutics for this disease.

The traditional Chinese medicine (TCM) is an extraordinary national treasure and benefits human health worldwide (Dashtdar et al. 2016). Herbs are increasingly recognized for their therapeutic value against variety of human diseases including cancer. *Herba Epimedii* (Chinese name: Yin Yang Huo) has historically used in China to nourish the kidney and strengthen the bone (Zhang et al. 2008), and other potential therapeutic benefit has been extensively exploited in different disease models both *in vitro* and *in vivo*. Composition analysis of *Epimedii* has identified baohuoside-I (also known as icariside II) as the major active flavonoid component of *Herba Epimedii*, which possesses a broad-spectrum of pharmacological activities including anti-osteoporosis, anti-inflammatory, antioxidant and neuroprotective effects (Wu et al. 2013). More recently, baohuoside-I have been demonstrated that capable of inducing apoptosis in human non-small-cell lung cancer cells via reactive oxygen species-related mitochondrial signalling (Song et al. 2012) and inhibiting the growth of U266 multiple myeloma (Kim et al. 2011), which indicated potential and invaluable therapeutic benefit of this traditional medicine in cancer management.

MicroRNAs are a class of small oligonucleotide widely expressed in nature and play critical physiological roles in mammals (Ambros 2004). Through post-transcriptional modulation of target gene expression, microRNAs fine tune the complex signalling network underlying cellular behaviour. Assembling evidences indicate active involvement of microRNAs in each facet of tumour biology such as incidence, metastasis, resistance and recurrence (Ohtsuka et al. 2015). In this study, we attempted to interrogate the antitumour potential of baohuoside-I against melanoma and elucidate the underlying molecular mechanism, we concentrated on the alteration of microRNAome in response to baohuoside-I and pursued to understand its involvement in the potential therapeutic effect of baohuoside-I.

## Materials and methods

### Cell culture

Human melanoma cell lines M14 and MV3 were obtained and authenticated by the America Type Culture Collection (ATCC). Cells were maintained in RPMI-1640 medium supplemented with 10% foetal bovine serum (Hyclone, Waltham, MA) and 1% PSG (penicillin-streptavidin-glutamine). The log phase cells were cultured in humidified incubator at 37 °C with 5% CO_2_ supply. Transfection was performed with Lipofectamine 2000 (Invitrogen, Carlsbad, CA) according to the manufacturer’s instructions.

### MTT assay

Cell viability was assessed with MTT assay kit (CellTiter 96 Non-Radioactive Cell Proliferation Assay, Promega, Madison, WI) according to the supplier’s instructions. Briefly, equal amount of exponentially growing cell was seeded into 96-well plate for 24 h culture and subjected to either mock or indicated treatment. Add 10 μL MTT solution per well to archive a final concentration of 0.45 mg/mL and incubate 1∼4 h at 37 °C. Another 10 μL solubilization solution was added into each well to dissolve formazan crystals. Culture plate was vigorously vibrated to ensure complete mixture and solubilization. Absorbance at 570 nm was recorded on micro-plate reader (BioTek, Winooski, VT).

### Transwell migration assay

The cell invasion assay was performed with Transwell chamber (BD, San Diego, CA). Cells were harvested and re-suspended in serum-free medium and laid on the top of polycarbonate Transwell filter pre-coated with Matrigel (BD, San Diego, CA). The lower compartment was filled with complete culture medium containing 10% foetal bovine serum. After 24 h of consecutive culture, the noninvasive free cells were cautiously removed with cotton swab from the top chamber. The invaded cells in Matrigel were fixed first with 4% paraformaldehyde and visualized by crystal violet staining. Cell number was counted in every five random fields under microscope to evaluate invasive capacity.

### MicroRNA microarray assay

Total RNA was extracted using Trizol reagent (Invitrogen, Pleasanton, CA). The quality and quantity was first determined by Bioanalyzer 2100 (Agilent, Santa Clara, CA) prior to any further processing. The relative expression of microRNAs was assessed by Affymetrix GeneChip miRNA 2.0 arrays (Affymetrix, Santa Clara, CA) in accordance with manufacturer’s instruction. Briefly, 250 ng of total RNA was polyA tailed and labelled with biotin with the FlashTag Biotin HSR RNA Labeling Kit (Genisphere, Hatfield, PA). The hybridization was performed on rotation at 48 °C overnight and the array was scanned on the Affymetrix 3000 GeneScanner. Data were analyzed with the Affymetrix GeneChip Command Console software and relative signal intensity and normalization was generated by the Robust Multichip Analysis (RMA) algorithm. The miRNA candidates with > twofold change and *p* < 0.05 were considered as biologically significant.

### Real-time PCR

The candidate miRNAs were further validated by Q-PCR. 1 μg of total RNA was reversely transcribed with miScript Reverse Transcription Kit (Qiagen, Valencia, CA). Real-time PCR was conducted with miScript PCR Kit (Qiagen, Valencia, CA) on PRISM 7900HT. The relative expression was normalized to 5s rRNA and calculated by the 2^−ΔΔC^*^t^* method.

### Xenograft assay

Four-week old female B-NSG nude mice were purchased from Biocytogen and housed in pathogen-free environment. All experimental animal protocol was approved by the Institutional Committee of Animal Care and Use of Shandong Provincial Hospital affiliated to Shandong University. The indicated cells at exponential phase were harvested and suspended in sterile PBS. Single-cell solution was mixed with equal volume of Matrigel (BD) and subsequently inoculated subcutaneously into the right flank of recipient mice. Tumour growth was regularly monitored twice a week. Tumour volume was estimated according to the formula: TV (mm^3^) = length × width^2^ × 0.5. All mice were sacrificed at indicated time, tumour was stripped and measured.

### Dual luciferase reporter assay

TargetScan program was employed for prediction of target gene of miR-14. The 3′-UTR of candidate gene was cloned into luciferase reporter vector according to the provider’s instruction (Promega). Both M14 and MV3 cells were seeded into six-well plate the day before transfection. Co-transfection of luciferase reporter plasmid and miR-14 was performed with Lipofectamine 2000 (Invitrogen). Relative luciferase activity was measured by micro-plate reader (Molecular Devices, Sunnyvale, CA).

### Western blotting

Cell lysate was prepared in RIPA lysis buffer. The protein concentration was determined using BCA Protein Assay Kit (ThermoFisher, Waltham, MA). Equal amount of protein was resolved by SDS-PAGE electrophoresis and transferred to PVDF membrane. After blocking with 5% skim milk/TBST for 1 h, the membrane was hybridized with indicated primary antibody at 4 °C overnight. The PVDF was then washed with TBST buffer rigorously for 30 min and incubated with HRP-conjugated secondary antibody. The residue antibody solution was completely washed off with TBST, and protein band was visualized by enhanced chemiluminescence method (ECL, Millipore, Billerica, MA). GAPDH served as loading control for normalization.

### Clinical sample

Melanoma tumour sample were collected from the Shandong Provincial Hospital affiliated to Shandong University in accordance with the protocol approved by the Institutional Committee. Written informed consent was obtained from all patients enrolled in this study. Total RNA was extracted from liquid nitrogen preserved fresh tissue samples with Trizol method, and quality was checked by Bioanalyzer 2100 before use.

### Statistics

Data from at least three independent repeats were subjected to variance analysis using SPSS 23 (SPSS Inc., Chicago, IL), and were presented as Mean ± standard deviation (SD). The statistical significances between data sets were expressed as *p* values, and *p* < 0.05 was considered as statistically different.

## Results

### Baohuoside-I inhibits cell proliferation and migration in melanoma cells

**Figure 1.**
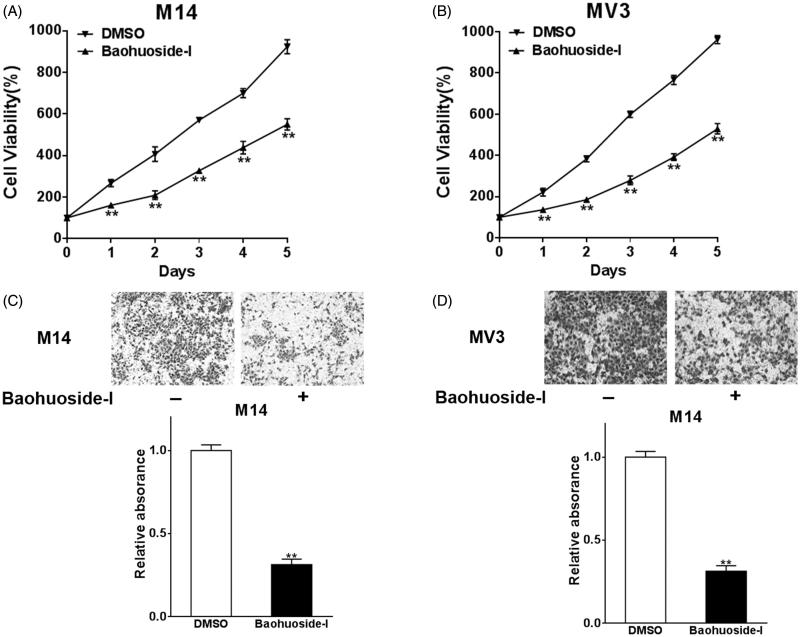
Baohuoside-I inhibits cell proliferation and migration in melanoma cells. M14 and MV3 cells were treated with 20 μg/mL baohuoside-I or DMSO. (A–B) The cell proliferation abilities were analyzed by MTT assay. Data were presented as mean ± SD from three independent experiments with triple replicates per experiment. **p < 0.01 compared to DMSO group. (C–D) Transwell migration assay was employed to analyze the migration abilities of the cells. Data were presented as mean ± SD from three independent experiments with triple replicates per experiment. **p < 0.01 compared to DMSO group.

First, we set out to investigate the inhibitory effect of baohuoside-I on malignant behaviour of human melanoma *in vitro*. Both M14 and MV3 cells were treated with mock or 20 μg/mL baohuoside-I dissolved in DMSO. The cell viability was measured by MTT assay. As shown in Figure 1(A,B), the baohuoside-I treatment elicited dramatic suppression of cell proliferation in both cell lines while compared with mock treatment. Furthermore, for MTT assay, we performed a dose-response study to define EC_50_ and Emax of baohuoside-I after a continuous incubation of 24, 48, 72 and 96 h, and found that baohouside-I indeed exhibited a dose-dependent inhibitory effect on M14 cells (Figure S1). Next, we attempted to determine the impact of baohuoside-I on invasive capacity, which underlaid the metastatic malignance of human melanoma. Trans-well assay was employed here to evaluate the invasive behaviour in response to drug treatment. The invaded melanoma cells in the Matrigel were staining with crystal violet and representative images are shown in Figure 1(C,D). In addition to its inhibitory effect on cancer cell proliferation, baohuoside-I treatment caused approximate 70% reduction in respect to invasion capacity. Our data unambiguously demonstrated that *in vitro* treatment of melanoma with baohuoside-I elicited significant inhibition against both cell proliferation and invasion.

### Baohuoside-I up-regulates miR-144 expression levels

**Figure 2.**
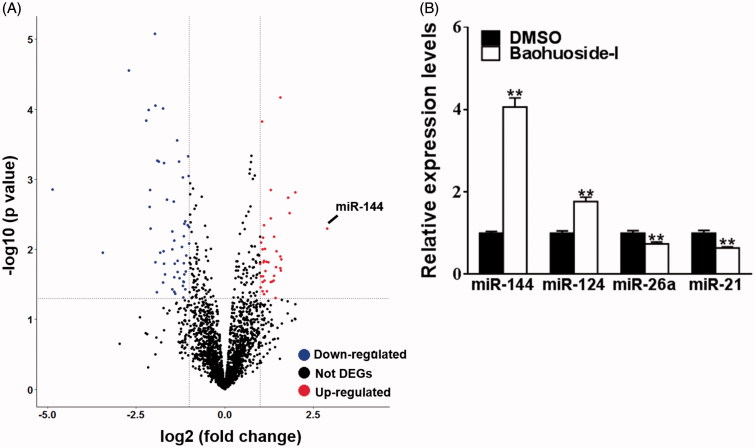
Baohuoside-I upregulates miR-144 expression levels. M14 was treated with 20 μg/mL baohuoside-I or DMSO. (A) After 24 h, the RNAs of the cells were isolated and were analyzed by microarray, and the different expression genes (DEGs) were displayed as volcano plot. (B) Cells were treated as above, the expression levels of differential miRNAs (miR-144, miR-124) were detected by qRT-PCR. Data were presented as mean ± SD from three independent experiments with triple replicates per experiment. **p < 0.01 compared to DMSO group. **p < 0.01 compared to DMSO group.

Next, we sought to elucidate the molecular mechanism underlying antitumour activity of baohuoside-I. We concentrated on the expression profile alterations of microRNA in view of the critical role of microRNA in malignance development. The Affymetrix Multispecies miRNA 2.0 Array was employed here to interrogate differentially expression miRNA in response to baohuoside-I treatment. The stringent threshold (fold change >2 and *p* < 0.05) was applied for confidential identification of potential candidate microRNAs. Totally 39 microRNAs were up- and 53 was down-regulated by baohuoside-I treatment, among which miR-144 showed the most remarkable increase (fold change >6) (Figure 2(A)). Moreover, our real-time PCR validated the results from discovery phase in several selected microRNAs including miR-144, miR-124, miR-26a and miR-21 (Figure 2(B)). Baohuoside-I treatment induced about four times increase of miR-144 expression in comparison with DMSO group, which might indicate a crucial role of miR-144 in mediating the antitumour activity of baohuoside-I against melanoma.

### Silence of miR-144 reverses the inhibition of baohuoside-I in melanoma

**Figure 3.**
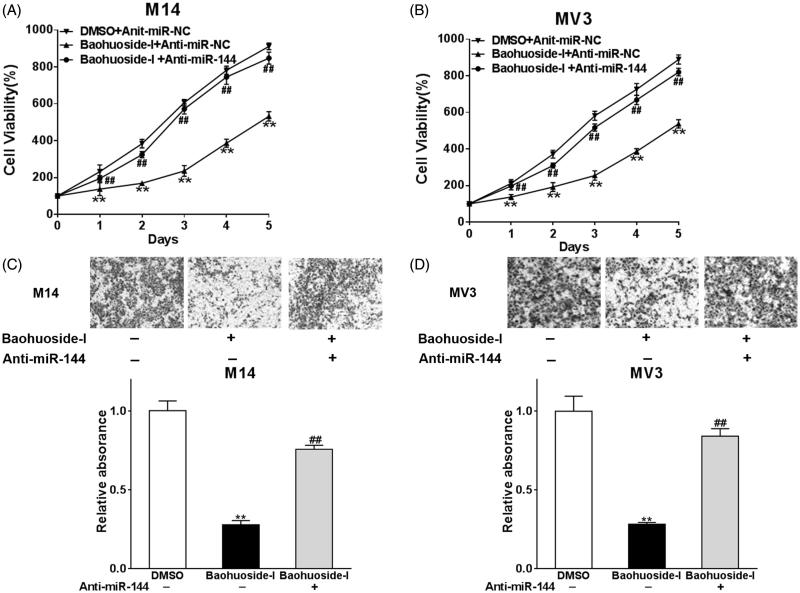
Silence of miR-144 reverses the inhibition of baohuoside-I in melanoma. M14 and MV3 cells were transfected with Anti-miR-144 or control (Anti-miR-NC), and then were treated with 20 μg/mL baohuoside-I or DMSO. (A–B) The cell proliferation abilities were analyzed by MTT assay. Data were presented as mean ± SD from three independent experiments with triple replicates per experiment. **p < 0.01 compared to DMSO + Anti-miR-NC group. ##*p* < 0.01 compared to baohuoside-I + Anti-miR-NC group. (C–D) Transwell migration assay was employed to analyze the migration abilities of the cells. Data were presented as mean ± SD from three independent experiments with triple replicates per experiment. **p < 0.01 compared to DMSO + Anti-miR-NC group. ##*p* < 0.01 compared to baohuoside-I + Anti-miR-NC group.

Our previous results demonstrated that the expression of several microRNAs was stimulated upon baohuoside-I treatment, which might underlaid its antitumour activity. We further investigated whether miR-144 specifically involved in baohuoside-I elicited proliferation inhibition and invasion suppression. As shown in Figure 3(A,B), cell viability compromised by baohuoside-I was nearly completely restored by miR-144 knockdown in both melanoma cell lines. Similarly, the density of invaded population as surrogate of invasion capacity dramatically increased in miR-144 knockdown cells while challenged by baohuoside-I (Figure 3(C,D)). Our data suggested that up-regulation of miR-144 in response to baohuoside-I treatment definitely and predominately contributed to its antitumour activity.

### SMAD1 is the new target of miR-144

**Figure 4.**
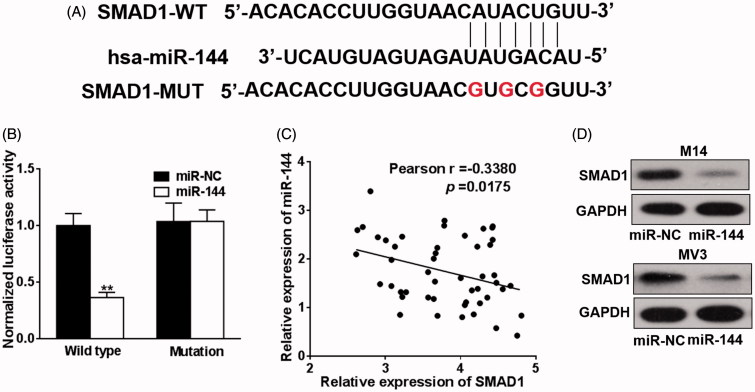
SMAD1 is the new target of miR-144. (A) TargetScan program was used to predict the targets of miR-144, and the putative seed-matching sites or mutant sites (the letter “G” in the 16th, 18th and 20th position) between miR-144 and 3′-UTR of SMAD1 according the program. (B) Luciferase reporter assay was employed to the luciferase activities of the WT and mut reporters. Data were presented as mean + SD from three independent experiments with triple replicates per experiment. **p < 0.01. (C) miR-144 and SMAD1 expression levels of melanoma specimens (*n* = 49) were analyzed by qRT-PCR and the relationship between which was tested by Pearson’s method. (D) M14 and MV3 cells were transfected with miR-144 or control mimics (miR-NC). After 48 h, the expressions of SMAD1 were detected by western blotting.

As aforementioned that endogenous miR-144 was subjected to modulation by baohuoside-I treatment and specific knockdown of miR-144 abrogated the inhibitory effect of baohuoside-I in melanoma cell lines. Accumulating evidence suggested that microRNAs actively played role in tumourigenesis, metastasis and drug resistance development via post-transcriptional regulation mechanism. Next, we should identify and validate the biological target gene of miR-144 and attempt to understand the molecular events downstream miR-144 induction. The online available bioinformatics tool TargetScan was employed here for the predictive purpose. As shown in Figure 4(A), we have identified prefect matched seed region in the 3′ UTR of SMAD1 with highest comprehensive score. Dual luciferase reporter plasmid carrying either wild-type or seed region mutated 3′ UTR of SMAD1 was co-transfected with miR-144 mimic to examine the potentially regulatory function. Our result demonstrated around 60% reduction of luciferase activity was caused by ectopic co-expression of miR-144 mimic (Figure 4(B)) in comparison with scramble sequence. The endogenous protein level of SMAD1 was determined in both cell lines upon transfected with miR-144 mimic, which was consistent with luciferase reporter results (Figure 4(D)). Moreover, clinical sample collected from melanoma patients manifested a reverse correlation between miR-144 and SMAD1, which further consolidated our *in vitro* data (Figure 4(C)). Our data suggested that SMAD1 was novel target of miR-144 in this setting.

### Baohuoside-I suppresses melanoma *in vivo*

**Figure 5.**
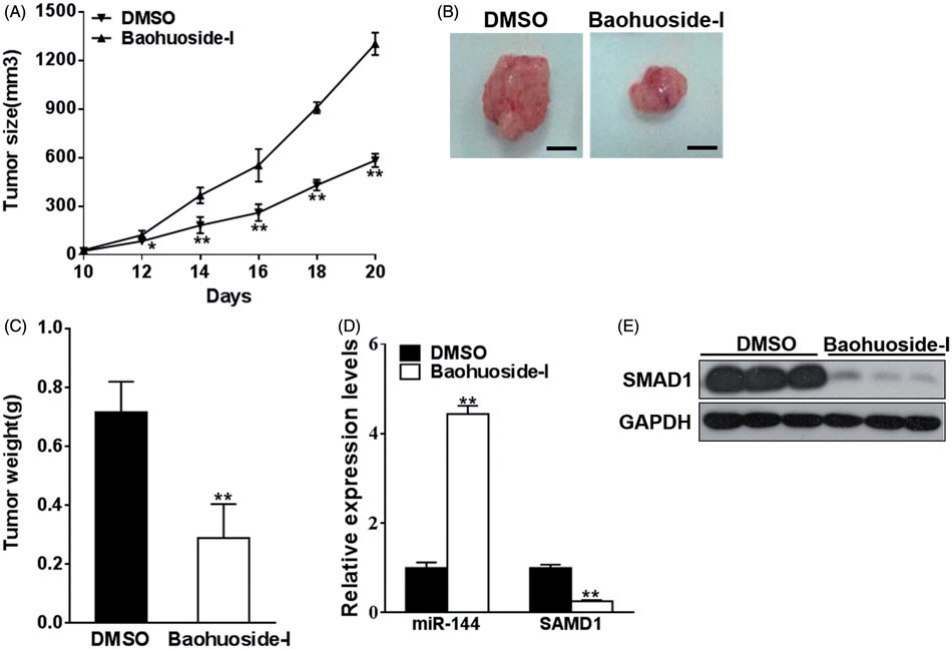
Baohuoside-I suppresses melanoma *in vivo*. M14 cells were resuspended in serum-free RPMI-1640 medium and were injected into each side of posterior flank of the nude mice, and the mice were treated with baohuoside-I (25 mg/kg) or DMSO by tail vein injection every two days. (A) The tumours were measured every two days. Data were presented as mean ± SD from eight tumours. **p < 0.01 compared to DMSO group. (B–C) In day 20 the tumours were stripped, photographed and weighed. Data were presented as mean ± SD from eight tumours. **p < 0.01 compared to DMSO group. (D) The RNA expression levels of miR-144 and SMAD1 were analyzed by qRT-PCR in the tumours. Data were presented as mean ± SD from eight tumours. **p < 0.01 compared to DMSO group. (E) The protein expression levels of SMAD1 of the tumours were tested by western blotting.

Our previous *in vitro* results demonstrated that baohuoside-I inhibited melanoma cell proliferation and invasion via up-regulation of miR-144 and in turn down-regulation of SMAD1. In view of the potential therapeutic value of baohuoside-I in melanoma, here we sought to further investigate *in vivo* antitumour activity of baohuoside-I. Xenograft nude mice were established by subcutaneous inoculation of M14 cells. Mice were treated with either baohuoside-I or mock via tail vein injection. As shown in Figure 5(A), tumour growth curve drawn from estimated tumour size demonstrated significant antitumour effect of baohuoside-I. In the representative images of excised tumour from sacrificed mice, actual tumour from baohuoside-I-treated group was much smaller than DMSO group (Figure 5(B,C)). Body weight of mice was also monitored during the treatment, and none of general animal toxicity was observed for baohuoside-I with the dosage adopted in our investigation (Figure S2). Moreover, the expression of both miR-144 and SMAD1 was determined in xenograft tumour samples. In agreement with *in vitro* data, miR-144 was significantly induced by baohuoside-I, which in turn decreased the expression of SMAD1 at both transcriptional and translational level (Figure 5(D,E)). In addition, we also found that the expression level of SMAD4 was not altered by the treatment of baohuoside-I (Figure S3). Our xenograft results correlated with the *in vitro* finding and highlighted the potential clinical value of baohuoside-I in melanoma patients.

## Discussion

Baohuoside-I is the active medicinal component extracted from *Herba Epimedii*, which possesses great therapeutic value in the treatment of variety human diseases such as sexual dysfunction, osteoporosis and cardiovascular disease. Besides, other potential clinical benefits of baohuoside-I have been extensively exploited so far. Notably, in the past decade, accumulating evidences indicated the antitumour activity of baohuoside-I in broad-spectrum of human malignances. For instance, baohuoside-I treatment was demonstrated to induce apoptosis in human non-small cell lung cancer via modulation of reactive oxygen species-mediated mitochondrial pathway (Song et al. 2012). Baohuoside-I also displayed inhibitory effect on hypoxia-inducible factor-1α, which underlaid its antitumour activity against osteosarcoma (Choi et al. 2008). Meanwhile, the alternative mechanism has been proposed by another lab that blockade of EGFR/mTOR pathway resulted in the inhibition of cell proliferation in osteosarcoma (Geng et al. 2014). In prostate cancer cells, activation of cyclooxygenase-2/prostaglandin E2 pathway has been suggested to mediate baohuoside-I induced apoptosis (Lee et al. 2009). The JAK2/STAT3 pathway activation has been demonstrated that involving in baohuoside-I elicited cell death in U266 multiple myeloma cells (Kim et al. 2011). In malignant melanoma, combination of paclitaxel with baohuoside-I potentiated the apoptosis-inducing effect by inhibiting TLR4 pathway (Wu et al. 2012). In addition, baohuoside-I treatment elicited cell-cycle arrest via ROS-p38-p53 signalling as well (Wu et al. 2015). In line with these previous reports, here we demonstrated that baohuoside-I treatment significantly compromised cell viability of two independent human melanoma cell lines. Besides, our data also suggested that invasion capacity has been dramatically suppressed by baohuoside-I. Our data confirmed the antitumour potential of baohuoside-I in melanoma. With aid of microRNA array, we further characterized the microRNAome in response to baohuoside-I treatment. Notably, miR-144 was one of the most dramatically stimulated genes in our results.

Cumulative evidence supported the tumour suppressor function of miR-144. Ectopic introduction of miR-144 mimic significantly inhibited hepatocellular carcinoma cell proliferation, invasion and migration by targeting ZFX (Bao et al. 2017). The gastric cancer progression was markedly suppressed by miR-144-3p elicited inhibition of epithelial-to-mesenchymal transition (EMT) via modulation of PBX3 (Li et al. 2017). In breast cancer, miR-144 functioned as a tumour suppressor through inhibiting ZEB1/2-mediated EMT process (Pan et al. 2016). Similarly, miR-144 served as critical suppressor gene in renal cell carcinoma by targeting MAP3K8 pathway (Liu et al. 2016). The diagnostic indicative value of miR-144 has also been extensively interrogated. For example, the elevated expression of miR-144-3p was evaluated as a noninvasive biomarker for the acute myeloid leukaemia with exclusive targeting NRF2 (Sun et al. 2017). The significant upregulation of miR-144 in serum samples of oesophageal cancer patients was considered as the minimally invasive marker (Sharma et al. 2016). And content of circulating miR-144 along with other 5 miRs in breast cancer patients has been demonstrated in association with progression-free survival and early detection of metastasis in this disease (Madhavan et al. 2016). Solid data from different groups indicated the intimate association between miR-144 and the malignant behaviour such as invasion, migration and metastasis. Consistent with these observations, here we provided evidences in support of the critical role of miR-144 in mediating baohuoside-I imposed antitumour effect on melanoma. Relative expression of miR-144 in both M14 and MV3 cell line was significantly stimulated by baohuoside-I in our study. Moreover, the predominant role of miR-144 in baohuoside-I elicited suppressive effect on melanoma was further demonstrated by manipulating its expression. Efficient knockdown of endogenous miR-144 almost completely abrogated the proliferation inhibition and invasion suppression induced by baohuoside-I.

Next, we further pursued the pathological role of miR-144 through identification of its candidate target. Based on the bioinformatics prediction, we confirmed SMAD1 as novel target of miR-144 in this setting. SMAD1 belongs to the SMAD family, which are signal transducers and transcriptional modulators that mediate multiple signalling pathway. SMAD1-mediated signalling cue from bone morphogenetic proteins (BMPs) ligand, which was subsequently phosphorylated and formed complex with SMAD4 as functional transcription regulator (Liu et al. 1996). SMAD1 pathway is involved in diverse biological processes such as cell growth, apoptosis, morphogenesis, development and immune modulation. The activation of ACVR2/SMAD1/SMAD4 played fundamental role in autocrine bone morphogenetic protein-9 mediated ovarian cancer cell proliferation (Herrera et al. 2009). The SMAD1 induction by BMP signalling, along with up-regulation of MMP-2 was critical for pancreatic cancer invasiveness (Gordon et al. 2009). In line with these notions that SMAD1 functioned as oncogene involving in promotion of cancer cell growth and invasion, we have unambiguously demonstrated that expression of SMAD1 was significantly suppressed in response to baohuoside-I treatment, which exclusively depended on miR-144 up-regulation. Moreover, our xenograft mice manifested optimal response to baohuoside-I administration, which highlighted the potential of clinical application of the natural chemical.

In summary, we have exploited the potential therapeutic effect of baohuoside-I against melanoma both *in vitro* and *in vivo*. The up-regulation of miR-144 and thus suppression of SMAD1 might eventually contributed to this antitumour activity of baohuoside-I.
